# Root causes of underperforming urban waste services in developing countries: Designing a diagnostic tool, based on literature review and qualitative system dynamics

**DOI:** 10.1177/0734242X221074189

**Published:** 2022-02-08

**Authors:** Hans Breukelman, Harold Krikke, Ansje Löhr

**Affiliations:** 1 Faculty of Management, Science and Technology, Open University of the Netherlands, Heerlen, The Netherlands; 2 Department of Environmental Sciences, Faculty of Science, Open University of the Netherlands, Heerlen, The Netherlands

**Keywords:** Solid waste management, developing countries, diagnosis tool, qualitative system dynamics, governance, cities

## Abstract

Cities in developing countries struggle with providing good waste collection services to all their citizens. Daily practice mostly shows low service coverage, especially in the poorer parts of cities. Up until now, research has mainly dealt with the symptoms of poor performance. This article aims at designing a qualitative System Dynamics model of the urban system that may serve as a diagnostic tool to find the root causes and leverage points for interventions. The research presented here uses a broad literature review to draw up a complex causal loop diagram describing all relevant urban variables (demographic, economic, social, financial, technical and governance-related) and their relations. The diagram is analysed using qualitative methods, partly derived from graph theory. It results in an evaluation of all variables, paths, loops and branches of the model, and finally in a simplified model. This simplified model is helpful in diagnosing waste management problems in cities, in formulating interventions and their points of leverage and even in formulating a new taxonomy that classifies cities with regard to the effect and delay in their urban processes. When it comes to interventions, the model suggests that the root cause is in populations growing faster than their economies, and that the enabling circumstances are mainly in poor governance practices that are unable to secure that tax incomes keep pace with needed budgets for sound services.

## Introduction

Solid Waste Management (SWM) is considered a crucial aspect of public services as it directly affects the health and well-being of citizens, the recovery of resources and the quality of the environment. As yet, the original concern of SWM, being to remove inhabitants’ waste from the vicinity of urban housing, remains a problem in many Cities in Developing Countries (CDCs) with many of them showing citizens being serviced by waste collection at less than 50% (Kaza et al., 2018; Wilson et al., 2015).

Breukelman et al. (2019) conclude that research into this problem has mostly focused on symptoms whereas underlying reasons have attracted little attention. The field lacks diagnostic tools that may help to analyse causes and improve interventions. These authors also infer that the performance of waste collection in CDCs can be looked at as the product of a complex urban system in which processes on demographics, economics and the physical (infra)structure interact with social and political processes and public services. Interactions are numerous and often show time-delays and feedbacks. They conclude that System Dynamics (SD) can be in a good position to provide for such a diagnostic tool. The method deals with dynamic, complex systems by drawing up qualitative causal models of the relations between variables, gauging these relations and ultimately capturing them in mathematical equations, checking the overall model on adequacy and using the model to find root causes and simulate the impacts of interventions (Forrester, 1969; Morecroft, 2015; Sterman, 2000). Many disciplines have shown the method’s helpfulness (Bala et al., 2017; Pejic Bach et al., 2019). This study is the first to apply SD to describe the interactions between demographic, economic, governance, financial and technical variables relevant to a city’s performance on waste collection.

Although many researchers value SD for its quantitative promise, there is a growing group that pays attention to the intermediate stage of qualitative modelling and qualitative analysis. At the core of this stage are Causal Loop Diagrams (CLD) and Stock Flow Diagrams (SFD). Both enable to study the structural complexity of systems before stepping up to investigate their dynamic complexity through simulations (Liebovitch et al., 2020). Qualitative analysis tries to assess CLDs and SFDs in order to improve model-adequacy, evaluate the importance of individual subsystems, variables and their relations, to remove redundancies and to hypothesise on expected behaviour and useful interventions (Hürlimann, 2009; Liebovitch et al., 2020; Oberlack et al., 2019; Wolstenholme, 2004). Qualitative SD has shown to be especially useful in addressing very complex systems, systems with not-quantifiable variables and systems that lack the availability of data sets (Beck et al., 2012; Mirchi et al., 2012; Murphy and Jones, 2020; Schoenenberger et al., 2014).

We will do the CLD-modelling in four sections, followed by sections ‘Discussion’ and ‘Conclusion’. In the first section titled ‘Literature-based system description’, we will use a literature review and expert opinions to describe the most important urban processes that may influence the target variable, being the performance on waste collection and city cleaning. Based on this description, the second section ‘Model’ will define these processes in terms of variables and their causal relations and aggregate them in a first draft of the CLD. In the third section titled ‘Qualitative analysis’, we will qualitatively analyse the model by assessing the individual variables, the paths that connect them to the target variable, the loops in the model that cause feedbacks inside the system and the branches that integrate the effects of multiple variables. In the fourth section titled ‘Simplifying the model’, we will try to simplify the model by eliminating exogenous and non-essential variables and using the results of the third stage.

## Literature-based system description

A literature review was performed for providing the needed system description. The review was performed during March to December 2020. Sets of keywords, congruent with the clustering, were used as entry, followed by forward and backward snowballing, and resulted in a corpus of 81 references. Given the broad field of covered disciplines, the review does not pretend to be exhaustive nor error-free. We contend, however, that it is usable at this point. The results of this review will be discussed in three clusters being (1) urbanisation and economy, (2) governance, finance and public interaction and (3) SWM.

### Urbanisation and economy

Urbanisation and economic growth seem to go hand in hand (Glaeser, 2011; Henderson, 2010). Urbanisation leads to agglomeration benefits (Ahrend et al., 2017; Kennedy et al., 2011), pulling more inhabitants to cities. On the other side, increased agricultural productivity pushes rural inhabitants to migrate to cities (Bidandi, 2015; Varis, 2006). Economic growth can lead to a reduction of poverty when at the same time inequality declines (Bhorat et al., 2016).

More recent studies for developing countries indicate that, while economic growth seldom comes without urbanisation, urbanisation does not automatically cause economic growth (Castells-Quintana, 2017; Henderson, 2010). Many countries in Sub-Saharan Africa reveal urbanisation without economic growth (Bhorat et al., 2016; Gross and Ouyang, 2021; Varis, 2006). A demographic explanation is that urbanisation is the combined result of in-migration and natural population growth. Where in-migration may be pulled and absorbed by economic growth, natural growth is not. High natural growth may thus erase or balance out the positive relation between urbanisation and economic growth (Castells-Quintana, 2017; Gross and Ouyang, 2021; Mangi et al., 2020; Mariathasan, 2016). A more economic line points the finger at low ratios of industrialisation (Dercon et al., 2019; Venables, 2017). Besides that, more exogenous factors like civil unrest and natural disaster can play a role (Bhorat et al., 2016; Bidandi, 2015; Calleja et al., 2017; Castells-Quintana, 2017; Meagher, 2010; Phonphoton and Pharino, 2019; Rice and Rice, 2012).

Nevertheless, in many CDCs, the combined effect of (some) economic growth and continued urbanisation propels an increase of build-up area, population density, traffic and consumption that is much faster than ever witnessed in any developed country (Henderson, 2010; Mangi et al., 2020; Varis, 2006). In many cases, and with regard to many urban services, this growth outpaces the capacity of administrative organisations to adapt themselves and their cities (Bettini et al., 2015; Bidandi, 2015; Hill and Lindner, 2010; Mariathasan, 2016; Mason et al., 2020; Rice and Rice, 2012). This imbalance can be referred to as ‘Urbanisation Overhang’ or ‘Overurbanisation’ (Rice and Rice, 2012).

Where this happens in an urban context with low institutional quality (Beard et al., 2016; Henderson, 2010; Levitsky and Murillo, 2009; Varis, 2006), fragmented governance (Ahrend et al., 2017; Cheema et al., 2010; Haas and Wani, 2019; Horen, 2004), discontinuities in management (Ezeah and Roberts, 2014), absence of stable leadership (Cheema et al., 2010; Cornea et al., 2017; Heymans et al., 2016; Yeboah-Assiamah et al., 2014), tax incomes and urban investments start to dwindle (Arimah, 2005; Rammont and Amin, 2010; Rice and Rice, 2012). And in turn, this leads to diseconomies of agglomeration (Ansah, 2010; Dercon et al., 2019; Haas and Wani, 2019; Venables, 2017) initiating a vicious circle (Beard et al., 2016; Dercon et al., 2019), also called the Malthusian trap (Castells-Quintana, 2017; Rice and Rice, 2012).

Once the path of slum formation and urban sprawl is initiated, it becomes increasingly more difficult to revert (Dercon et al., 2019; Mangi et al., 2020). Weak formal institutions and poor urban services then lead to lower public trust (Güemes, 2019; Hirschmann, 1999).

In these situations, the informal sector flourishes (Kubanza and Simatele, 2018; Oguntoyinbo, 2012). Prices charged by the informal sector are often higher than those of formal services (Heymans et al., 2016) and their productivity is low, thus adding to an overall lower efficiency of the city (Bhorat et al., 2016; Varis, 2006).

In larger countries with a number of competing cities, the relative attraction of a city would then go down and rural migration would shift to other cities (Beck, 2012; Castells-Quintana, 2017; Wolff et al., 2020). But in smaller countries with few cities, this shift is hindered. Meanwhile the push of rural migration continues and natural population growth only goes down very slow (Gross and Ouyang, 2021; Varis, 2006; Wolff et al., 2020).

### Governance, finance and public interaction

In situations where the above processes coincide with (decentralisation in) a weak governance setting, a breeding ground is created for corruption and clientelism. These processes mostly favour middle- and upper-class parts of the city (Bidandi, 2015; Chattopadhyay, 2017; Cheema et al., 2010; Coetzee, 2015; Cornea et al., 2017; Dercon et al., 2019; Devas, 2001; Ezeah and Roberts, 2014; Güemes, 2019; Heymans et al., 2016; Hirschmann, 1999; Horen, 2004; Jones et al., 2014; Kennedy et al., 2011; Kubanza and Simatele, 2018; Meagher, 2010; Michelutti and Smith, 2014; Tang and Huhe, 2016; Yeboah-Assiamah et al., 2014), thus increasing inequality and reducing economic growth (Rodríguez-Pose and Zhang, 2019). It accelerates growth of poverty to a pace faster than the population growth, a phenomenon known as Urbanisation of Poverty (Beard et al., 2016; Horen, 2004; Rice, 2014). Sometimes deliberate abuse of public power is accelerating this process (Dercon et al., 2019; Henderson, 2010; Lei and Green, 2017; Tang and Huhe, 2016).

The combined effect of urbanisation overhang, urbanisation of poverty, weak governance and corruption is that only wealthier citizens have access to essential public services and it reduces public trust (Cheema et al., 2010; Kinyondo and Byaro, 2019; Wittemyer et al., 2014). Public trust and support is to a great extent the result of a gap between expectations and actual performance (Güemes, 2019; Kang and Park, 2018). Awareness, here defined as the perceived importance of having access to services, leads to growth in expectations and in the willingness to participate in public services (Berdi, 2019; Cheema et al., 2010; Flores-Macías, 2018; Kang and Park, 2018; Kinyondo and Byaro, 2019; Shao et al., 2018). Besides that, public trust is also affected by the quality of governance in general (Calleja et al., 2017; Cheema et al., 2010; Güemes, 2019; Levitsky and Murillo, 2009; Ochoa and Graycar, 2016; Sulemana, 2015; Tang and Huhe, 2016; Wittemyer et al., 2014; Yeboah-Assiamah et al., 2014; Zhong, 2014).

A low willingness-to-pay for urban services makes them vulnerable to restricted funding through the general budget (Beard et al., 2016; Flores-Macías, 2018; Jones et al., 2014). This process is worsened in case of poor governance quality (Bonilla and Zapparoli, 2017; Cheema et al., 2010). Fiscal decentralisation may strengthen institutional quality and financial autonomy if well taken care of (Beard et al., 2016; Bonilla and Zapparoli, 2017; Dercon et al., 2019; Mason et al., 2020; Sohail et al., 2005). Healthy finances at the local level can be improved by applying earmarked taxes (Felgendreher and Lehmann, 2016; Flores-Macías, 2018; Haas and Wani, 2019; Kubanza and Simatele, 2018; Rammont and Amin, 2010). Similarly, sound operations can best be managed in separate organisations with some autonomy (Alam, 2016; Felgendreher and Lehmann, 2016; Heymans et al., 2016; Jones et al., 2014; Kennedy et al., 2011; Sohail et al., 2005).

A good tandem of institutional quality and economic growth seems to be at the basis of finding a way up, out of this deadlock (Arimah, 2005; Beard et al., 2016; Bonilla and Zapparoli, 2017; Castells-Quintana, 2017; Dercon et al., 2019; Devas, 2001; Haas and Wani, 2019; Heymans et al., 2016; Hirschmann, 1999; Levitsky and Murillo, 2009; Mariathasan, 2016; Rodríguez-Pose and Zhang, 2019).

### SWM

The availability and quality of urban services are to a large extent the result of the above processes, and SWM services are no exception. SWM performance, here defined as the ability of city authorities to collect municipal waste on a daily basis and in an equitable way, is challenged by growing volumes of generated waste due to both population and economic growth (Beigl et al., 2008; Estay-Ossandon and Mena-Nieto, 2018; Ghinea et al., 2016; Grazhdani, 2016; Intharathirat et al., 2015; Kaza et al., 2018; Majale, 2011). By character, waste collection is a logistical process and its performance is ruled by available budgets, road infrastructure, traffic density, inhabitants participation, and planning and managing skills (Kaza et al., 2018; Wilson et al., 2015).

When a city is not able to provide its citizens with waste collection, also here, informal actors jump in (Horen, 2004; Majale, 2011; Minja and Mbura, 2019; Oguntoyinbo, 2012; Solomon, 2011). Services provided by informal collectors may look beneficial as it provides services at no cost for urban authorities and leads to some form of recycling (Tinio et al., 2019). The downside is that these services are often partial, unsystematic, discontinuous and expensive (Haregu et al., 2017; Horen, 2004; Kaza et al., 2018; Kazuva et al., 2018; Wilson et al., 2015). Some cities involve community-based organisations and non-governmental organisations (NGOs) in their services but also this has downsides such as fragmentation, increased costs, diminished control, inefficiency at the city-level and deepening inequality (Horen, 2004; Sohail et al., 2005).

Slum-areas are in general not or poorly serviced because of weak governance and poor accessibility (Jones et al., 2014) and, as a result, slum-populations are often not willing to pay any contribution (Boateng et al., 2019; Horen, 2004; Majale, 2011; Rammont and Amin, 2010). Affordability may not be the reason for this lack of willingness as also cities in poor countries have the scale to provide SWM services to all citizens at a reasonable price (Batool and Ch, 2009; Breukelman, 2019; Devas, 2001; Felgendreher and Lehmann, 2016; Huisman et al., 2016).

Evacuating the waste out of the city by formal SWM services needs stable cash flows (Horen, 2004) and growing cities can benefit from their economies of scale (Carvalho and Marques, 2014; Lombrano, 2009; Santibañez-Aguilar et al., 2013) but also here there are agglomeration problems. City growth implies that waste needs to be transported over longer distances in heavy traffic and transfer stations are needed (Fugii, 2019; Kinobe et al., 2015; Rathore and Sarmah, 2019; Thikimoanh et al., 2015). If this is not done properly, the efficiency goes down quickly (Rathore and Sarmah, 2019).

## Model

SD has been used in a variety of SWM-related studies (Bala et al., 2017; Pinha and Sagawa, 2020; Popli et al., 2017). An important tool used in most SD studies is the CLD. A CLD gives an abstract and qualitative description of the system-processes (combinations of variables and their relations).

CLD drawings use a convention on symbols and notation (Sterman, 2000). The relations between variables are given as arrows, representing the causal direction. A + or −sign, added to this arrow, means that an increase in the causing variable leads to an increase or decrease respectively in the affected variable. An = sign added to the arrow indicates that a process is slow or delayed. A causal loop is defined as a set of variables that feed back to each other. A CLD can hold many loops. If a loop has an uneven number of negative relations, the loop is balancing (indicated as a B); an increase in a variable eventually leads to a correction that brings down that same variable again. If the number of negative relations is even, the loop is called reinforcing (indicated as an R). Loops with delays give the system their dynamic behaviour.

We used the processes that emerged in section ‘Literature-based system description’ above, to draw up the CLD. For this, we considered that the CLD should describe a generalised urban system with the system boundary, being the city boundary, including these processes. Exogenous variables (outside this system boundary) may influence the dynamics in the city but not (only negligibly) vice versa. Some endogenous variables (inside the system boundary) may also be considered as exogenous but only if they are not influenced by other endogenous variables.

**Figure 1.**
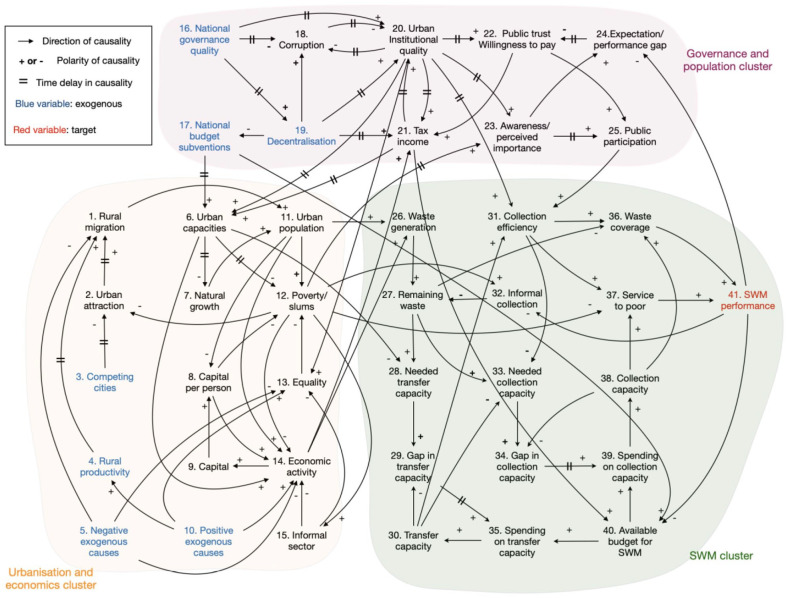
First draft causal loop diagram integrating all variables and relations with regard to governance (green), urbanisation (yellow) and SWM (purple).

SWM performance is used as the target variable for this research and is defined as the composite functioning of the city with regard to coverage of waste services and the availability of this service to poor inhabitants. The system description and a first draft of the CLD were shared with six experts in the field of urbanisation, sustainability and SD. Based on their comments we adapted the model, resulting in the CLD presented in Figure 1.

The CLD displays the three clusters, used in the system description. This consolidated diagram comprises a total of 41 variables which are described in Appendix 1. The number of exogenous variables is 7. Between the variables, there are 87 connecting relations.

## Qualitative analysis

The qualitative analysis will use tools, derived from Graph Theory (Browning, 2015; Oliva, 2004). They have been applied on a wide variety of topics, such as research on peace-processes, terrorism, innovation, management and water resources, over the last 15 years (Beck et al., 2012; Hürlimann, 2009; Liebovitch et al., 2020; Schoenenberger et al., 2014). With the help of these tools, complex CLDs can be turned into matrices that are easier to handle and investigate. There are a number of variations in this type of analyses. For this article, we use the following methods and adapt them to our needs:

- Variable analysis: the so-called cross-impact and cross-time matrices (CIM and CTM) are constructed. Both are square matrices, that take the variables from a CLD and use them both as column- and as row-numbers. For those variables in the CLD that have direct causal relations to each other, the corresponding cells are filled with a weighting for impact and time. The matrices are then used to assess the importance of every individual variable towards its directly neighbouring variables in the system.
- Path analysis: using the same square matrices, this method evaluates the effect of all individual variables on a target variable (in this case variable 41: ‘SWM performance’). The number of paths from each variable to the target variable is calculated. Together with the weighting on delay and impact, it provides insight in the relative importance of each variable for influencing the target variable.
- Loop analysis: the matrices are now used to count and group all feedback loops that include the target variable and evaluate what happens to these loops when individual variables are taken out of the system. If deletion of a variable leads to a decline in the number and effects of these loops, this may be used as an indication that this variable is important for the target variable.
- Branch analysis: in this final method, the paths that reach the target variable are traced backwards. This backtracking into the branches is limited to a certain number of steps. All variables that are encountered on this number of backward steps are counted. Variables that appear frequently in these branches may be of great influence on the target variable.

The four types of analysis can be considered as complementary. Where variable analysis studies the behaviour of a point (variable), path analysis does so with a line (linear strain of variables), loop analysis with a circle (variables in a loop) and branch analysis with a network (branched variable connections).

### Variable analysis (VA)

For this analysis, we translate the CLD into a CIM and CTM. The CIM is constructed and weighted as follows:

- The CIM is a square matrix (also called adjacency matrix) with 41 × 41 cells and each row/column presenting one of the 41 variables.
- A value in cell CIM_*ij*_ means that variable *i* is causing an effect on variable *j*. No value means there is no causal relation between the two.
- If the value is positive, it means that an increase in variable *i* causes an increase in variable *j*; if the value is negative, it means that an increase in variable *i* causes a decrease in variable *j*.
- The values themselves represent the authors’ weighted estimate of the relative strength or effect of the relations. A value of 1 means that the increase or decrease is proportional, a value of 2/3 means it is sub-proportional and a value of 3/2 suggests that the relation is over-proportional. This type of weighting and its attribution to the relations in the CLD is subjective and not based on any quantitative underlying information. It is derived from weighting methods used in other studies (M. Beck et al., 2012). Using these values has an advantage in path analysis, as will be explained there.

The resulting matrix is shown in Appendix 2. The last column calculates the combined strength of all outgoing relations (the Active Sum or AS) of variable *i* as described in equation (1)

(1) $${\mathrm{AS}}_{i}=\overset{41}{\underset{j=1}{\sum }}\left|{\mathrm{CIM}}_{ij}\right|$$

The AS can be regarded as a relative measure for the influence of a variable on its neighbouring variables. One could consider to use the arithmetic mean instead of the sum but that could underestimate the influence of those variables that have multiple, small effects.

Likewise, the aggregated effect of all incoming relations (the Passive Sum or PS, see last row) on variable *j* can be calculated as in equation (2)

(2) $${\mathrm{PS}}_{j}=\overset{41}{\underset{i=1}{\sum }}\left|{\mathrm{CIM}}_{ij}\right|$$

The PS is a relative indication of the extent in which a variable is influenced by others. From Appendix 2, we may conclude that, considering the assumed strengths, Urban Institutional Quality (variable 20) can be considered as the most influential variable and Economic Activity (variable 14) as the one that is most influenced.

In a similar way, the CTM is constructed and weighted:

- A value in cell CTM_*ij*_ of this matrix means that variable *i* is producing a delay in the relation with variable *j*. Again, no value means there is no causal relation between the two.
- All values in this matrix are positive meaning that only delays (and no accelerations) are considered.
- The values themselves represent the authors’ estimate of the relative produced delay (PD) in the relations. A value of 1 means that there is no delay; the effect is immediate. A value of 2 means that the reaction is expected to be delayed on the short term (2 years). A value of 5 indicates a delayed effect of 5 years and a value of 10 means an effect after a long-term of 10 years. Also this weighting follows similar approaches in other studies (Beck et al., 2012; Hürlimann, 2009; Schoenenberger et al., 2014). Still, they must be regarded as subjective.

The resulting matrix is shown in Appendix 3. In this case, aggregation using the arithmetic mean is preferred instead of using the sum. Using a sum of all delays caused by a variable would not be a logical representation of delay as delays are in real years. Based on this weighting, we can add the delay in all outgoing relations (the PD, see last column) of variable *i* as in equation (3)

(3) $${\mathrm{PD}}_{i}=\frac{\left[\overset{41}{\underset{j=1}{\sum }}\left({\mathrm{CTM}}_{ij}\right)\right]}{n}$$

where *n* is the number of outgoing delays for variable *i*. Likewise, the aggregated delay of all incoming relations (the Received Delay or RD, see last row) on variable *j* can be calculated as equation (4)

(4) $${\mathrm{RD}}_{j}=\frac{\left[\overset{41}{\underset{i=1}{\sum }}\left({\mathrm{CTM}}_{ij}\right)\right]}{n}$$

where *n* is the number of incoming delays for variable *j*.

**Figure 2.**
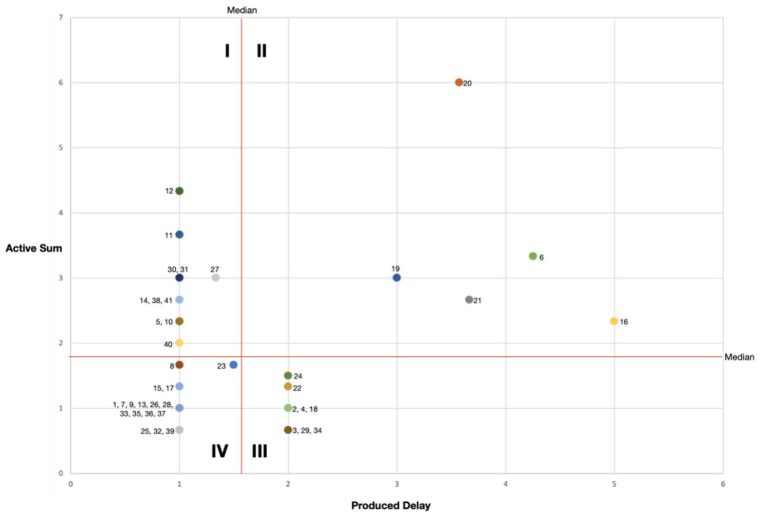
Plot of active sum against produced delay for all variables in the CLD of Figure 1.

AS and PD describe the impact of a variable within (a part of) a system. Impact may be understood in multiple ways, depending on the type of problem. Variables with a high and fast impact may seem important, but in certain cases, a low and slow impact may be more critical, especially in systems where inertia is the problem, as, for example, in many problems on urban governance. Whatever the problem, a plot of AS against PD may reveal important information. For the system in this article, this is done in Figure 2.

The plot is divided into four quadrants separated by boundaries being the median values of AS and PD. Quadrant I variables act as conductors or distributors; they typically pass on impulses directly and are impactful towards their neighbours. We see that prevalence of poverty and slums (12), population (11), economy (14) and the remaining waste to be collected (27) fall in this category and that seems understandable. Also, variables that directly relate to the capacity of waste management processes (30, 31, 38 and 40) are in this Quadrant which may imply that processes around these variables tend to be important but not limiting. Quadrant II variables act as capacitators as they are able to slow down potentially impactful processes. Most variables in this Quadrant relate to governance aspects, with urban institutional quality (20) being the most important. They make important processes slow meaning that they may lead to a kind of passivity towards their direct surroundings, or even of the system as a whole. Quadrant III variables work as insulators; they slow down processes and reduce their output. The variables in this quadrant seem to relate to processes at the level of individual inhabitants, such as trust (22), expectation (24) and attraction (2) but also to corruption (18) and gaps that need to be addressed (29 and 34). Quadrant IV variables act as indifferent components or even as resistors. They do not delay processes but just relay and/or reduce them. There are 17 variables in this category and they come from all clusters in the CLD. Many of them can be regarded as mere intermediates that may be removed from the CLD without effect on the performance of the model.

This variable analysis indicates that public governance processes are influential but slow. They may cause inertia in their surrounding subsystem. Whether they also affect the behaviour of the system as a whole, and SWM performance in particular, cannot be concluded through this type of analysis. Variables relating to urbanisation appear to propel the system whereas variables on SWM processes do not seem to play an inhibiting role.

### Path analysis (PA)

The variable analysis is not capable of assessing a variable’s impact on the system as a whole nor on a specific variable. For this, path analysis may provide a better option. A path is defined as a sequence of variables that connects a start- and an end-variable while allowing any other variable to be in the path only once. The analysis can be done in a number of ways, for example, by tracking the shortest path between both variables and then calculating the consolidated polarity, impact and delay of this causal path. As there may be many paths between two variables, it may be more meaningful to assess all paths between the two and calculate the consolidated effect of all these paths. Making such an inventory in a system with 41 variables needs automation and can be done using an algorithm that searches through the CIM and CTM matrices. For this, we used an existing algorithm (Hürlimann, 2009) and adapted it to calculate the effect of all individual variables on the target variable SWM Performance (41). The algorithm discriminates between paths that have an overall positive or negative polarity. For each variable *i*, it calculates the following characteristics of every path from variable *i* to variable 41:

- NumPP_*i*_ and NumNP_*i*_ being the number of all positive and all negative paths, respectively.
- AvLenPP_*i*_ and AvLenNP_*i*_ being the average length of all positive and all negative paths, respectively. Length is here defined as the number of variables visited in a path.
- AvEffPP_*i*_ and AvEffNP_*i*_ being the average path-effect of all positive and all negative paths, respectively. Path effect is defined as the arithmetic product of the effects (CIM_*ij*_) of all individual variables of a path. Here, the use of weights 2/3, 1 and 3/2 shows the advantage referred to above as it makes the path effect independent of path depth.
- RaEff_*i*_ being the ratio AvEffPP_*i*_/(AvEffPP_*i*_ + |AvEffNP_*i*_|). It is used as a measure for the overall positive effects on SWM performance. If RaEff_*i*_ of variable *i* is larger than 0.5, then the overall effect is positive (and vice versa).
- AvDelPP_*i*_ and AvDelNP_*i*_ being the average delay of all positive and all negative paths, respectively. Delay is defined as the sum of all delays (CTM_*ij*_) caused by individual variables of a path.
- AvDel_*i*_ being the average of AvDelPP_*i*_ and AvDelNP_*i*_. It is used as a measure for the overall delay in the effects of variable *i* on SWM performance.

**Figure 3.**
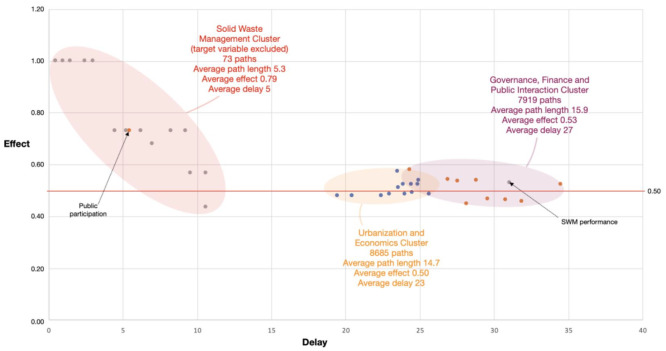
Plot of RaEff_*i*_ against AvDel_*i*_ for all variables in the CLD of Figure 1.

Figure 3 shows a plot of the average path effect (RaEff_*i*_) against average delay (AvDel_*i*_) for each variable’s impact on SWM performance.

The figure allows for some observations. A first one is that a total of almost 17.000 paths lead from all 40 variables to the target variable 41. This high number is typical for similar, highly branched and feed-backed systems (Schoenenberger et al., 2014).

A second observation is that SWM variables, especially on waste collection and service coverage (30, 31, 35, 38, 39 and 40), seem to be fast conductors of impulses towards the target variable and that these impulses are conveyed with low attenuation. This is logical as they are close to the target or even part of the target variable (36 and 37). But, in part, it is also the character of the processes taking place in this SWM cluster which are more or less operational.

At this point, the influence of path length must be emphasised. The cluster on SWM variables has only few feedbacks, and as a result, the number of paths and the average path length are low when compared to the other clusters. For these low path lengths, the used system of weighting and processing in the algorithm may lead to overemphasising effects and underestimating delays.

The average effect of variables in the Urbanisation and Economics cluster is at 0.50 indicating that the positive and negative effects from these variables are more or less in balance or indifferent. Nevertheless, when taking a closer look, it appears that variables in this cluster that relate to population growth and poverty (variables 1, 2, 3, 4, 7, 11, 12 and 13) have a negative impact on the target variable. Economy variables (6, 8, 9 and 14) have a positive impact meaning that growth in these variables leads to improved SWM performance.

The variables in the cluster on governance, finance and public interaction appear to have the slowest impact albeit with slightly more effect than those in the urbanisation cluster. Here also a distinction can be made between variables relating directly to the quality of governance and availability of budget (16, 17, 20, 21 and 22) that have a positive relation to SWM performance, and variables as corruption and decentralisation (18 and 19) that do the opposite, which is in line with section ‘Literature-based system description’. Variables on expectations and awareness (23 and 24) also have a negative impact which is logical because growing awareness tends to widen the expectation gap which, in turn, erodes public trust. Public participation (25) is an interesting outlier as its influence is more or less similar to variables in the SWM cluster. Looking at the place and connections in the CLD, the variable may indeed be looked at as a result instead of as a part of the governance cluster. In that sense, it could just as well be categorised under the SWM cluster.

The target variable SWM performance (41) also dispatches paths itself (to be exactly: 500) that eventually return to the variable itself. On average, this feedback is slow but positive, meaning that the variable tends to reinforce itself.

### Loop analysis (LA)

**Figure 4.**
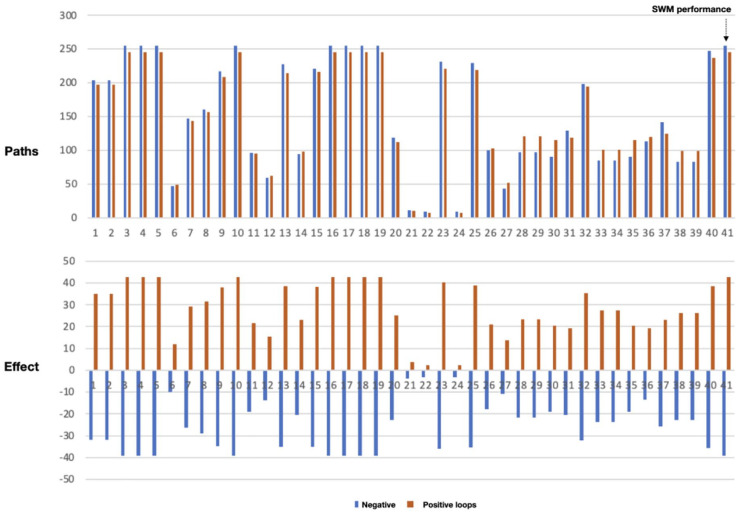
Plot of remaining loops (number in upper graph and effect in lower graph) that include variable 41, after deleting individual variables from the CLD of Figure 1.

Loop analysis studies the importance and behaviour of loops in a network. Loops can be important because they may influence the dynamics of a system through their feedbacks, especially when combined with delays. In highly connected networks, loop analysis may therefore better describe the system’s behaviour than path analysis. For our analysis, we used the algorithms from path analysis and made the start- and end-variable the same. When applied to the target variable SWM performance (no. 41), the program finds 245 positive loops and 255 negative loops. The program is then run repeatedly, and in each run, one variable is deleted from the system while the other 40 are remaining. The result is shown in Figure 4 in which the *X*-axis gives the variable-number that is deleted. The bars in the upper part of the graph provide the number of remaining negative (blue) and positive (orange) loops that still include the target variable 41. The lower part presents the overall effect of the remaining positive and negative loops.

A trivial outcome of this graph is that deleting exogenous variables (3, 4, 5, 10, 16, 17 and 19) does not affect the target variable as these exogenous variables are by definition not a part of any loop. Another observation is that deleting variables related to tax income (21), public trust/expectation (22 and 24) and urban capacities (6) leads to a near breakdown of the number and effect of all loops going through variable 41. Also, deleting variables on population (11), equality (12) and economy (14) has strong effects. This may imply that all these variables play an important role in influencing the target variable.

With the exception of informal collection (32), practically all SWM variables, when deleted, lead to halving the effects on the target variable. This group-wise behaviour is also apparent around population variables (6, 7, 11, 12, 26 and 27), economy variables (6, 8 and 14) and governance (20, 21, 22 and 24).

When looking at the ratio between positive and negative paths, we see that the effect of positive paths gets a little more important when deleting the variables urban capacities (6) and remaining waste (27). The opposite appears when deleting public trust (22) and awareness (24). There is no obvious explanation for these effects.

Surprisingly, deleting corruption (18) has little to no effect which could imply that it is not an important variable in the many loops leading to the target variable. Deleting the variable available budget (40) does not seem to affect the number of paths but, all the same, it reduces the overall effect on the target variable.

### Branch analysis (BA)

**Figure 5.**
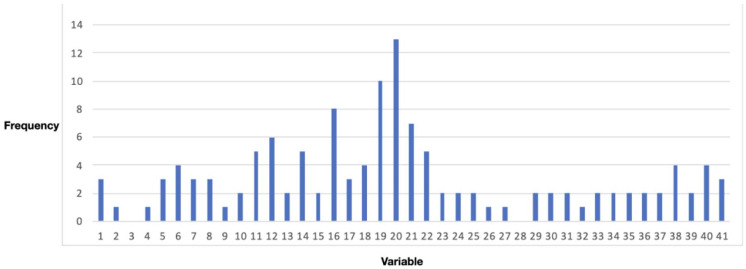
Frequency of appearance of variables on paths leading to the target variable 41 in Figure 1, using a path depth of five relations.

This part of the analysis walks back the paths from the target variable. In a first step going back from variable 41 in Figure 1, we encounter variables 36 and 37. Going one step further, variables 12, 27, 31 (twice) and 38 (twice) are encountered. An algorithm is used to explore these paths up to a depth of five connecting relations and count the frequencies with which variables are found in these relations. The result is given in Figure 5.

This analysis also shows that especially those variables that relate to governance aspects (16, 19, 20, 21 and 22) play an important role in the paths leading to the target variable. Variables on economy (6, 8 and 14) and population (11 and 12) also show a high frequency. When looking at the SWM variables, collection capacity (38) and budget (40) score above average.

## Simplifying the model

This section tries to answer the question whether the complexity of the CLD of Figure 1 is really needed for providing an accurate and useful description of the system’s behaviour. This question is prompted by the observations that (1) a number of variables act as indifferent transmitters of incoming impulses (VA, PA and LA) and that (2) many variables show group-wise behaviour (VA, PA, LA and BA). The assessment results therefore invite to simplify the CLD of Figure 1 as much as possible and justified.

There may be several ways to simplify the model. One is to remove all exogenous variables from the CLD. This ‘endogenisation’ does not deny the existence and influence of exogenous variables; it is merely done to enable focusing on endogenous variables only, a fundamental starting point of SD. Endogenisation can be followed by eliminating those intermediate variables that do not contribute to the system’s behaviour. This can be done by including their role in the incoming and outgoing relations (encapsulation). Bureš (2017) describes a procedure for this approach. The encapsulation starts with deleting the most basic variables (Single-Input-Single-Output, SISO), followed by elimination of DISO variables (Double-Input-Single-Output) and so on with SIDO (Single-Input-Double-Output), TISO (Triple-Input-Single-Output) and SITO (Single-Input-Triple-Output) variables until a satisfying simplicity has been achieved. The method aims at consolidating the system into a limited number of MIMO (Multi-Input-Multi-Output) variables. The limitation of such an approach is, however, that at some stage, two MIMO variables are connected through relations that are not readily understandable and sometimes even contradictory (one MIMO has both positive and negative direct causal relations with the other MIMO). Resolving such contradictions leads to choices that may fundamentally affect the system’s behaviour.

Another method is to use the group behaviour of related variables to aggregate them into one new variable. McGlashan et al. (2016) show the use of this modularity method to detect structural clusters in complex CLDs. Here also, there is a risk of making choices that affect the system as a whole, but this risk may be minimised using the results of a preceding analysis such as the one in this article.

**Figure 6.**
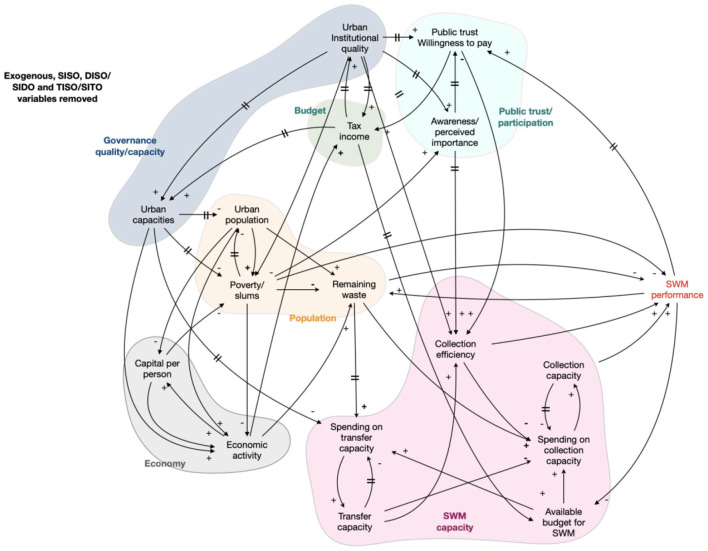
Intermediate result of simplifying the CLD of Figure 4 through eliminating exogenous variables and encapsulating SISO, DISO, SIDO, TISO and SITO variables.

Using Bures’ method, up to encapsulating TISO/SITO variables, leads to a simplified model as given in Figure 6. It is important to notice that this simplification does not lead to any change in the system’s expected behaviour as only intermediate variables are deleted up to a level where no contradictions have to be resolved.

**Figure 7.**
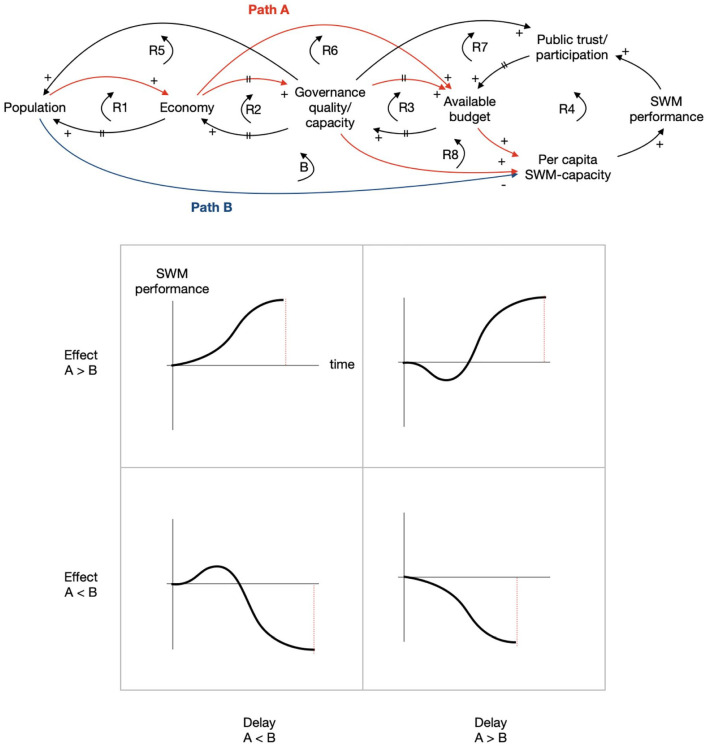
Projections of SWM performance under different circumstances with regard to effect and delay in the dominant paths.

The coloured fields in Figure 6 represent groups of variables that revealed, in the qualitative analyses above (Figures 3–5), a similar behaviour and a similar effect on the target variable. We used this phenomenon to further simplify the model by aggregating such a group into one variable. This final step results in the model depicted in Figure 7.

On the left-hand side, it depicts the slow but influential processes on governance, budget, economy and population. They are related to each other by a set of five reinforcing loops. In such a situation, exogenous impulses may trigger virtuous or vicious cycles resulting in effects such as urbanisation without economic growth, urbanisation overhang and poverty trap as mentioned in section ‘Literature-based system description’.

Population growth, however, shows an important side effect as it leads to more waste and more inhabitants to be serviced, thus reducing the adequacy of SWM capacity. This produces the only balancing loop in the system. Budget, capacity, performance and participation are in a reinforcing loop with little delay.

The expected behaviour of this system may either be one of continued growth or decline in SWM performance. Whether it is growth or decline may be strongly influenced by the pace of population growth. Figure 7 shows two important paths originating from population: a red Path A going through economy, governance quality/capacity and available budget towards SWM capacity and a blue Path B going directly to SWM capacity. One can imagine that the effects and delays in these paths dominate the resulting evolution of SWM performance over time. The plots in Figure 7 represent expected SWM performances under different regimes of effects and delays in Paths A and B. A high effect and low delay in Path A may be the most beneficial scenario for SWM performance. More delay in Path A will lead to an initial reduction in SWM performance that will gradually and in a longer period be overcome. A low effect and high delay in Path A do the opposite.

When looking for leverage points for interventions to improve SWM performance in this system, it is important to consider the following:

- The population and economy part of the system on the left-hand is not useful for interventions as these processes are autonomous, difficult to manage and any intervention may result in counter-productive effects. For example, reducing the influx of new inhabitants may seem attractive, but it will also negatively affect the economy and reduce available budgets.
- The SWM part of the system on the right-hand may appear to be a good point for interventions, but it is not. For example, any investment in capacity may lead to an increased performance on the short term, but if this is not followed by improvements in governance and yearly budgets, this upswing will not last long and may even spark decreasing public trust and a reluctancy to follow up on further improvements.
- The process of simplification leads to an aggregation of multiple variables in a limited number of consolidated variables. This does not imply that these underlying variables are no longer relevant when searching for leverage points. They retain their relevance, especially when further analysing routes of intervention.

The key to success is in the hands of governance. Political and administrative processes should focus on budget availability keeping pace with population- and city-growth. The most promising way to do so is to implement and enforce taxation schemes and meanwhile safeguarding service availability and quality in order to prevent eroding public trust.

## Discussion

The results of the analysis in this article must be treated cautiously. The model, the methods, the weighting, the algorithms and therefore also the results are vulnerable to subjective choices. The subjectivity that may be inherent in building the model is reduced using a large body of literature and having it reviewed by experts. It must, however, be acknowledged that the review is not exhaustive and may still lack essential insights. Therefore, this non-empirical method cannot lead to final conclusions on the model’s plausibility. The analysis may, however, still reveal whether the model adequately reflects the system description. As with regard to this, we involved the most important groups of relevant urban processes, that emerged from the literature, in the first draft of the model (Figure 1). The second draft of the model (Figure 7) loses on detail but still captures the essential urban processes described in the literature. This claim can be substantiated as follows:

- Urbanisation-without-economic-growth can still be the outcome of the simplified, second draft of the model. Population growth may lead to economic growth, but due to influences of exogenous variables and a possible negative feedback from inertia in governance processes, essential city processes may bring this economic growth to a standstill while, due to delays, population growth may still proceed for some time.
- Urbanisation-overhang is also described in the simplified model. If population growth is faster than the growth in governance quality, it leads to a decrease in the city’s capacity to supply services.
- The poverty-trap may start with a negative exogenous impact or with inertia in governance processes, bringing all the reinforcing feedbacks into a vicious, downwards cycle.
- Corruption-and-clientelism are, according to the literature review, important. Although we followed the literature and included these variables and their relations in the first draft of the model, the results of the qualitative analyses indicate that they are less significant than expected. It may be that the translation of literature into the first draft of the model is not adequate. Although we deleted the variable on corruption from the first draft, it is still incorporated under the aggregated variable of governance quality/capacity in the second draft of Figure 7.
- Public-support has remained an important item in the simplified model. It is represented by the variable public trust/support. The possible deadlock of eroding public trust, as mentioned in the literature, may arise when governance quality dwindles. Decreasing public trust could then bring the reinforcing loop R4 in Figure 7 into a negative spiral.
- Processes on logistics are consolidated under the variable ‘Per capita SWM capacity’ in Figure 7. Although simplified, especially with regard to the role of transfer stations, it still describes the positive impacts of available budget and governance quality and the negative impact of high population growth.
- Although informal waste collection (variable 32) is reported in the literature as being an important part of the system (Horen, 2004; Majale, 2011; Minja and Mbura, 2019; Solomon, 2011), the analyses show this variable to be indifferent with regard to the system’s behaviour. One reason for this outcome may be that the relations in the system of Figure 1 do not adequately describe reality. Another reason could be that informal waste collection is indeed important but at the same time transitional; it fills a gap for as long as formal services are inadequate and as long as informal workers use informal jobs as a survival strategy (Horen, 2004). In this sense, this found indifference may still be compatible with the literature.

With regard to the algorithms, the analysis uses choices on how ratios, sums and averages are made and on how deep paths are searched. These choices do not lead to deluding results as long as it is clear what these indicators represent. We tried some alternatives, but they did not influence the outcome in any major way. For example, raising the search depth in BA from 5 to 10 steps only made the resulting importance of variables more distinct. We therefore contend that the algorithms can be considered as useful for these analyses.

The weighting of effects and delays has, of course, an effect on the analyses’ results. Preferably, this weighting should aim at realistically reflecting the character of an individual relation and should also express proportionality towards other relations. For path analysis, we weighted effects using a number below or around 1 in order to prevent longer paths automatically leading to higher effects because of their multiplication. Of course, this also implies that using a weight smaller than 1 tends to reduce the effect of longer paths, but that may be realistic as one may expect that longer paths not only show more delay but also attenuation. Our choice of weighting the effects using 2/3, 1 and 3/2 is in line with weighting procedures used in other studies (Beck et al., 2012; Hürlimann, 2009; Schoenenberger et al., 2014).

The analyses do not give an indication that the level of complexity of the first draft is needed for the model’s usefulness. A number of variables are in the model merely because they define consecutive variables or because they explain the logic in the causal chains. As a result, they can be combined in one new variable as long as it is defined properly in the consolidated variable. Examples are the combination of waste generation (26) and remaining waste (27) and combining the target variable (41) with its composing variables (36 and 37).

Also, the results (especially those of path analysis) reflect a kind of group-like behaviour of variables as can be seen with variables on population/poverty (variables 1, 2, 3, 4, 7, 11, 12 and 13) and variables on governance/budget (16, 17, 20, 21 and 22). Reducing the number of variables by agglomerating them into new, further consolidated variables, is a logical choice when reducing complexity and it aligns with a similar approach used by McGlashan (McGlashan et al., 2016).

Although variable analysis does not produce insights in how the target variable of SWM performance is influenced by other variables, it does tell something about the overall dynamics in the system. It suggests that population growth acts as an engine of the system, governance as the slow driver and SWM processes as an immediate transmission. This system behaviour was also expected based on the literature review, for example, in the previous studies (Bidandi, 2015; Glaeser, 2011; Henderson, 2010; Mangi et al., 2020; Varis, 2006).

Path analysis relates every single variable to the target variable. We did not use the shortest path method, used, for example, by Beck et al. (2012), as this may not reflect accurately how a system works. Impulses in one variable in the system may have both negative and positive effects and choosing only one path could thus lead to a strong bias. Instead, we included all paths like is done by Schoenenberger et al. (2014). It shows that variables from the clusters in the first draft of the CLD indeed tend to aggregate in such a way that most SWM variables are fast and with high effects whereas urbanisation and governance variables are slow and with lower effects.

Loop analysis shows that deleting single variables from the urbanisation and the governance clusters leads to a near collapse of the importance of loops that include the target variable. This may imply that feedbacks make variables in loops much more important, or even make them key variables of the system. A similar result is found when looking at the results of the branch analysis. Based on this, it may be hypothesised that the effect of loops and branches dominate the behaviour of the system.

With all needed cautiousness, described above, the shared results of the four types of analysis imply that:

- The system-as-a-whole seems to work in the same direction as the targeted SWM performance, meaning that a positive SWM performance, when fed back into the system, results in an increasing positive development and vice versa.
- Population growth may be part of the root cause of underperforming SWM services. The variable has a strong impact and is in close reach of the target variable. Although paths leading from population growth to SWM performance show considerable average delay, this is probably due to the fact that population dynamics branches out its influence to many variables in the network and this may blur its fast effect on SWM performance.
- If this population growth also leads to economic growth the overall effect on SWM performance may still be positive. This is, however, very much dependent on the actual quality of urban governance. Governance processes tend to be influential but slow. When working well, they have a positive direct effect on SWM performance as well as an indirect positive effect via economic growth. When not working well, the opposite happens. Governance quality and capacity can therefore be regarded as an enabling circumstance for both positive and negative developments.
- SWM processes themselves do not seem to attenuate or slow down SWM performance.

These results are compatible with the literature review meaning that both the first draft and the second draft of the model are usable representations of reality. The analysis has improved the process of simplifying the model by complementing the method proposed by Bureš (2017). The complexity is vastly reduced by going from 41 to 7 variables.

The simplicity of the model, and the resulting quarters in the plot of Figure 7, may inspire thoughts on designing a taxonomy; a systematic classification of urban circumstances using the simplified model along with its most important parameters (effect and delay). Before doing so, the model needs calibration against existing data sets and testing in case studies.

## Conclusion

This article deals with poor performing urban waste management services in developing countries. Currently, the knowledge of this problem is mostly related to symptoms and not so much to the underlying causes and the way to diagnose them. This study uses SD to better understand the way economic, demographic, social, technical, financial and political processes interact, how strong/weak and fast/slow these interactions are, how they feedback and lead to reinforcements or inertia and how the system-as-a-whole turns into a cause. This way of looking at urban waste services is not done before and it opens up new ways to analyse and diagnose the problem.

This article describes the start of the design process for such a diagnostic SD model. This article shows the applicability and limitations of qualitative analysis methods for this purpose. We were able to design a simple CLD describing the most important processes. Based on this model, we projected the systems behaviour under different circumstances. The root cause for failing urban waste management services seems to lie in population growth, outpacing economic growth and the adaptive capacity of urban governance. Leverage points for interventions must be sought in the domain of governance, taxation and budgeting.

The simplified diagnostic model is useful to reduce complexity in describing processes relevant to urban waste collection in developing countries. Besides academics, it may help practitioners to gain oversight, focus on essential dynamics in the system and identify key leverage points. Notwithstanding this positive outlook, the model is based on the literature and expert review only and is tested nor calibrated using real world data. One road for further improvement is to invite the research community to challenge and improve the model. The other road should be one of quantitative modelling, testing and simulation and the application of the model in case studies. Eventually, the model may lead to some sort of taxonomy that may be useful when assessing a city’s problematic SWM performance.

## Figures and Tables

**Appendix 2.**
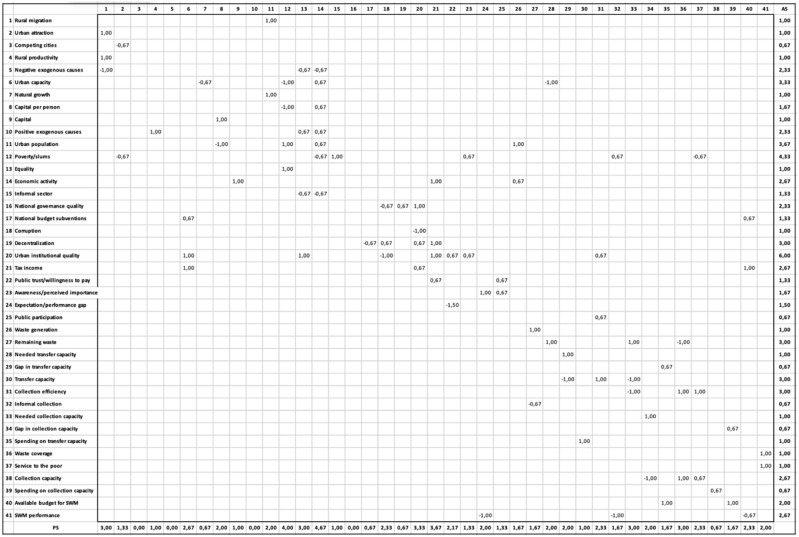
Cross-impact matrix of CLD in Figure 1.

**Appendix 3.**
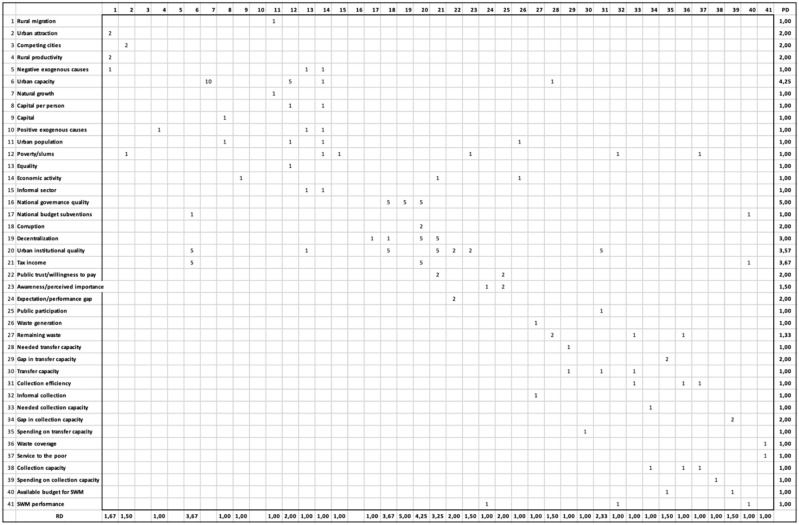
Cross-time matrix of CLD in Figure 1.

## Appendix

**Appendix 1.** Description of CLD-variables including the cluster they belong to and the number of ingoing and outgoing relations.

	Number	Variable	Description
	1	Rural migration	Number of rural inhabitants moving to the urban areas to settle there.
	2	Urban attraction	Relative attractiveness of the city when compared to other neighbouring cities.
	3	Competing cities	Attraction by other cities in the country (if any) in number and extent.
	4	Rural productivity	Rural products, produced per rural inhabitant.
	5	Negative exogenous causes	External variables outside the urban boundary such as climate change, natural disasters, ethnic fractionalization, unrest and war with a negative relation towards one or more endogenous variables.
	6	Urban capacity	Urban capacity to house, facilitate, transport and service present and incoming inhabitants.
	7	Natural growth	Population growth through birth and death rates.
	8	Capital per person	Capital accumulation per inhabitant per year.
	9	Capital	Capital accumulation in the city per year.
	10	Positive exogenous causes	External variables outside the urban boundary such as national economy, availability of natural resources, international prices and foreign aid with a positive relation towards one or more endogenous variables
	11	Urban population	Number of urban inhabitants.
	12	Poverty/slums	Prevalence of urban poverty and slums as percentage of inhabitants in this category.
	13	Equality	Prevalence of equality as reflected in income distribution.
	14	Economic activity	That part of the economy that registers with the government and pays taxes.
	15	Informal sector	Ratio of the informal sector as a percentage of all economic activities in the city.
	16	National governance quality	The quality of the national government in terms of transparency, capacity, legitimacy, allocation, pro-activity, authority, coordination, enforcement, stability and fairness.
	17	National budget subventions	That part of the urban budget paid for by contribution from the national budget.
	18	Corruption	Level of urban corruption and clientelism.
	19	Decentralisation	The extent of transfer of authority and responsibility from the national to the urban level.
	20	Urban institutional quality	The quality of the urban government in terms of transparency, autonomy, capacity, legitimacy, allocation, pro-activity, authority, coordination, enforcement, stability and fairness.
	21	Tax income	The total urban revenue income through taxation.
	22	Public trust/willingness to pay	Citizens’ confidence that political actors are producing outcomes consistent with their expectations, resulting in a positive inclination to pay needed taxes.
	23	Awareness/perceived importance	Public perception of the value of having access to public services on waste management.
	24	Expectation/performance gap	The divergence between citizens’ expectations towards public services and the way these expectations are actually fulfilled.
	25	Public participation	Willingness of citizens to take contribution as needed in waste collection by delivering their waste in the desired manner.
	26	Waste generation	The amount of waste that is produced by households and other economic actors and that is discarded of by them as having none or little value.
	27	Remaining waste	That part of the generated waste that is not collected through informal collection and awaits the services of formal urban service providers.
	28	Needed transfer capacity	Needed number and capacity of transfer stations that take the waste from collection vehicles and transfer it into bulk transport vehicles.
	29	Gap in transfer capacity	Divergence between needed and available transfer capacity.
	30	Transfer capacity	Available number and capacity of transfer stations that take the waste from collection vehicles and transfer it into bulk transport vehicles.
	31	Collection efficiency	The efficiency per unit of collection equipment and per worker in terms of tons, inhabitants and streets, including its adequacy to serve slum areas.
	32	Informal collection	That part of waste collection that is performed by the informal sector.
	33	Needed collection capacity	Needed number and capacity of collection equipment and workers who take the waste from the street and take it to transfer stations and disposal sites.
	34	Gap in collection capacity	Divergence between needed and available collection capacity.
	35	Spending transfer capacity	Total spending on capex and opex for transfer activities.
	36	Waste coverage	Percentage of generated waste, remaining after informal collection, that is collected and transferred out of the city through formal urban services.
	37	Service to the poor	Percentage of the poor urban population that has actual access to formal collection services.
	38	Collection capacity	Available number and capacity of collection equipment and workers who take the waste from the street and take it to transfer stations and disposal sites.
	39	Spending collection capacity	Total spending on capex and opex for collection activities.
	40	Available budget for SWM	Total budget available for capex and opex for collection and transfer activities.
	41	SWM performance	Composite target variable using waste coverage and service to the poor as a measure for SWM performance.

SWM: Solid Waste Management.

If light grey, exogenous (independent) variable or variable fully controlled by exogenous variables; if red, target variable; if yellow, variable in the urbanisation/economics cluster; if purple, variable in the governance/population cluster; if green, variable in the SWM cluster.
